# Time-aware forecasting of search volume categories and actual purchase

**DOI:** 10.1016/j.heliyon.2024.e25034

**Published:** 2024-01-19

**Authors:** Shahed Abdullhadi, Dana A. Al-Qudah, Bilal Abu-Salih

**Affiliations:** King Abdullah II School of Information Technology, The University of Jordan, Jordan

**Keywords:** Time series forecasting, Volume purchase, Actual purchase, Online market, E-commerce

## Abstract

The new e-commerce field has attracted businesses of all sizes, retailers, and individuals. Consequently, there is an ongoing necessity for applications that can offer predictions on trending products and optimal selling time. This research suggests aiding businesses in forecasting demand for various product categories by employing data mining algorithms on multivariate time series data. To ensure the most recent information, real-time data was gathered through APIs to build the first block in this research. While search volume was derived from the Keywords Everywhere tool, Amazon's search volume was derived from the Helium 10 tool and external features about actual purchased data. The harvested raw datasets went through multiple processes to generate the dataset and were validated. The models XGBoost, Linear Regression, Random Forest, long-short-term memory, and K-nearest neighbor were employed to predict the trends, and the performance is demonstrated using evaluation metrics, namely Mean Squared Error (MSE), Root Mean Squared Error (RMSE), Mean Absolute Error (MAE), and Coefficient of Determination (R2). Overall, Linear Regression outperformed, especially at a correlation coefficient of 0.9, with R2 = 90.688, MAE = 0.038, MSE = 0.003, and RMSE = 0.057. KNN outperformed on correlation coefficient of 0.7, R2 = 85.129, MAE = 0.045, MSE = 0.005, and RMSE = 0.068. XGBoost produced the best results with a correlation coefficient of 0.9, yielding R2 = 85.89, MAE = 0.042, MSE = 0.004, and RMSE = 0.062. Random Forest, on the other hand, achieves peak metrics with a correlation coefficient of 0.6, R2 = 84.854, MAE = 0.041, MSE = 0.004, and RMSE = 0.066.

## 1 Introduction

The world is experiencing explosive growth in information [1], leading to a persistent need for e-commerce [2] to find quick solutions that suit the rapid pace of life. E-commerce is oriented towards replacing traditional trading processes [3]. It stands out as one of the leading domains for businesses aiming to advertise their products or services across diverse industries [4]. Consequently, conventional enterprises strive to incorporate e-commerce into their operations to stay abreast of advancements [5]. The rapid growth of e-commerce is driven by the increasing preference for online shopping among individuals. This trend has captured the attention of retailers and decision-makers in both business and I.T. fields. To facilitate the decision-making process, accurate demand predictions are essential. Customers' demand can be derived from search and sales volume for a specific category. By combining both factors, an enhanced prediction can be obtained for both decision-makers and individuals in the retail business. External factors, such as occasions, sales events, or unexpected occurrences, can influence individuals' behavior.

Occasions such as holidays, Mother's Day, and the FIFA World Cup have an impact on individuals' purchasing behavior. For example, holiday retail sales in the United States were anticipated to reach around 942.6 billion U.S. dollars in 2022 [6]. Christmas is particularly significant from a business perspective, with individuals in the United States expected to spend between $943 and $960 billion in November and December of 2022. On average, consumers were projected to spend $883 on gifts and other holiday-related items [7]. Sales events, such as "Singles' Day” on 11/11, symbolize four ones, representing singles standing together. On this day, single individuals celebrate by indulging in self-gifting [8]. Originally observed in China, Singles' Day has now gained popularity in Southeast Asia and some European countries [9]. Retailers seized this opportunity to boost sales, with Alibaba, the Chinese e-commerce giant, offering deeply discounted merchandise for 24 h starting midnight on November 11, 2009, turning Singles' Day into a global commercial phenomenon [10]. Other notable sales events include Black Friday, the last Friday of November, and Cyber Monday, the Monday following Black Friday. In 2022, Shopify reported a record Black Friday Cyber Monday weekend sales of $7.5 billion from independent businesses worldwide, marking a 19 % increase from the $6.3 billion achieved in 2021 [11]. Unforeseen events, like the COVID-19 pandemic, significantly impacted sales forecasting, prompting a massive shift towards e-commerce globally.

Given these circumstances, retailers require precise sales demand information for informed decision-making. Accurate sales forecasting is crucial for reducing costs by avoiding excessive cash flow tie-ups, increasing profits through improved product sales, and preventing backlogs and inventory shortages. Insights into customer behavior derived from search volume and historical purchase data provide retailers with a clear vision of market trends to guide business development and achieve desired returns on investment (ROI). A comprehensive study defining fluctuating categories across months is key to this achievement.

This research addresses a real-life problem faced by retailers and business decision-makers, emphasizing the need for an application that predicts trending products and optimal selling times. The study proposes analyzing market trends by combining individual search volume with actual sales in diverse categories. When search volume increases in a specific category, it indicates the category's virality or trendiness, as discussed by Lunn [12]. Additionally, the research suggests a comparative study between datasets obtained from the Keywords Everywhere tool [13] (representing search volume) and Helium10 [14] (representing actual purchases). These tools were chosen based on criteria such as originality, data accuracy, and effectiveness, and data mining methods were employed for experimentation.

Subsequently, the research focuses on harvesting datasets through multiple APIs to establish a robust foundation. The datasets, spanning the last few years, aim to cover various scenarios for future predictions. The selection of dataset and test set size is meticulous for realistic results and efficient analysis. Time series analysis is applied to understand customer behavior at evenly spaced intervals, presenting data on a monthly time scale for optimal matching.

The research's novelty lies in the diverse datasets obtained from various APIs, including Keywords Everywhere and Helium 10 tools. It contributes significantly to understanding the relationship between sales volume, search volume, and other factors observed in experiments, elucidating how these factors influence search volume prediction. On a practical level, the research aids retailers in surviving and thriving by enabling accurate prediction of demand, strategic stock management, and efficient supply chain planning. Additionally, it empowers retailers to set competitive prices, offer targeted discounts, and execute effective promotional campaigns based on insights into future demand, thereby maximizing sales and profitability. The paper also highlights the importance of comparing different machine learning models to evaluate performance. This comparative analysis assists in determining which models should be further considered based on their ability to outperform others. The proposed time-aware forecasting framework integrates knowledge of customer behavior at distinct time scales.

The paper's structure is organized as follows: Section 2 discusses relevant seminal works, Section 3 outlines the proposed methodology, Sections 4, 5 report experimental results, and Sections 5, 6 present the discussion and conclusion.

## 2 Related works

Demand forecasting has captured the attention of retailers and researchers, as highlighted by Cheriyan et al. [15]. They emphasize the significant impact of accurate forecasting on the economic sector and aim to enhance future sales predictions. To achieve this, they employ machine learning algorithms, including the Generalized Linear Model (GLM), Decision Tree (D.T.), and Gradient Boost Tree (GBT). GBT yields the best results with 98 % accuracy. Wang et al. [16] explore the relationship between search volume and sales volume, investigating brand search rank, brand sales rank, brand search volume, and brand sales volume. Their experiments reveal a clear correlation between search volume and sales.

In contrast, Pereira et al. [17] propose a comprehensive approach to address challenges in both sales volume and supply-demand within the retailing supply chain management backdrop. They advocate for a predictive and adaptive management strategy for omnichannel retailing supply chains. E-commerce researchers often integrate time series into demand forecasting, as seen in the model by Palkar et al. [18], which aids retailers in decision-making regarding stock amounts to minimize costs. Similarly, Wu et al. [19] state that incorporating logistics into e-commerce workflows enables the prediction of future e-commerce order demand.

Chen et al. [20] study forecasts of retailers on Tmall, highlighting the difficulty of accurately estimating sales due to the diverse products and strong seasonal properties. They propose methods to improve forecasting performance, including extracting seasonality from groups of retailers. Jain et al. [21] apply forecasting techniques on superstore data, transforming the dataset from a daily to a monthly basis for better suitability. Abbasimehra et al. [22] describe demand prediction as foundational for planning activities, proposing a multilayer LSTM networks-based method for predicting highly oscillating data.

Niu et al. [23] propose a multivariate time series prediction method combining the XGBoost model with feature engineering, aiming to predict the next 28 days of each series with XGBoost achieving superior results with a 0.65 RMSSE. Salamai et al. [24] conducted a case study using a continuous Stochastic Fractal Search (SFS) method and Guided Whale Optimization Algorithm (WOA) to optimize the parameter weights of Bidirectional Recurrent Neural Networks (BRNN). Their algorithm achieves impressive results on a time series dataset from Kaggle. Kulshrestha et al. [25] propose the use of machine learning algorithms for advanced predictions and analysis, utilizing sales dataset segments from an e-commerce company.

### 2.1 Literature gap

Given the importance of accurate demand volume information, several studies have focused on product demand forecasting, whereby researchers have developed models to predict demand [18,21,22,26], while others have focused on predicting sales [27,28,29,30]. Additionally, some studies emphasized the significance of factors influencing sales, such as online reviews and search data [31] or customer search data with economic variables [32], as seen in Ref. [16]. However, the gap being addressed lies in the limited availability of comprehensive models that integrate diverse datasets, such as search volume and actual purchase data, to provide retailers with timely and precise insights for informed decision-making in the rapidly changing e-commerce environment.

This study addresses the gap by examining external factors influencing demand using multivariate data, combining search volume, sales volume, and other product features. The dataset is a crucial component, providing scope, robustness, and confidence in results. The dataset for this study is gathered from two different tools, Keywords Everywhere and Helium 10, for real-time data and unique features. To our knowledge, no previous research has combined these tools into one dataset to monitor the effect of these features on the prediction model. Recognizing the importance of time series in demand prediction, this research employs data mining methods in a time series manner, distinguishing it from approaches treating the problem purely as a regression problem [20,19], or emphasizing sales prediction as more of a regression problem than a time series [27].

## 3 Methodology

To enhance the quality of research, it is critical to follow the proper methodology, which serves as a road map for achieving specific research objectives [33]. This section will examine the methodological approach used in this research. In constructing the methodology, this research aimed to devise a comprehensive approach leveraging multiple methods' strengths. A methodological approach was developed as a result of a systematic exploration of available techniques, a literature review, and an empirical analysis. The suitability of each method for capturing different aspects of the data and problem domain was considered. Many papers shared a methodology and structure similar to this research, such as those referenced in Refs. [34,[35], [36], [37]].

**Fig. 1:**
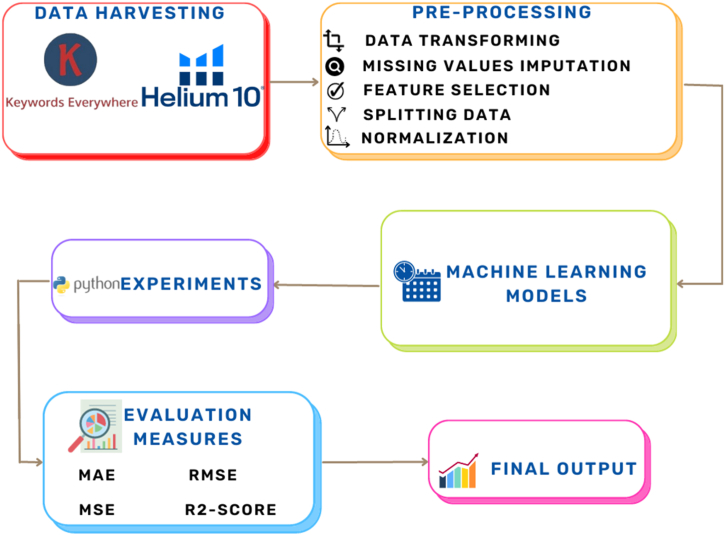
The proposed framework for experiments.

The proposed framework for this study is depicted in Fig. 1. In this section, we will elucidate the methodological processes undergone. The data mining models employed include XGBoost, Random Forest, LSTM, Linear Regression, and KNN. Evaluation measures encompass MAE, MSE, RMSE, and R2-Score.

### 3.1 Data collection

The rapid growth of the Internet and information technology has increased the accessibility of vast datasets, leading to a shift from seeking the best model to identifying the proper data for applying causal forecasting methods. This involves exploring variables that can impact future trends in specific markets. Traditional time-series forecasting often overlooks causal factors (independent features). This study aims to incorporate independent features into prediction models to address this gap.

The research focuses on the challenges that retailers encounter. The goal is to predict search volume for retailers, believed to directly impact sales, aligned with causal attributes. Careful selection of superior API tools is crucial, considering criteria such as data availability, visibility, relevance, and novelty. To the best of our knowledge, the chosen tools have not been utilized in similar research studies before. Consequently, data was collected using two API tools: the Keywords Everywhere tool and the Helium 10: X-ray tool.

The Keywords Everywhere tool has been employed in various data extraction domains, such as the work in Pirnau et al. [38], where the tool aided authors in identifying popular keywords and connecting them to Google users in relation to the analysis of blockchain technologies. Using this tool enabled the authors to develop “long-tail keywords,” facilitating the exploration of the correlation between internet users' searches and blockchain technology. Another study by Monett et al. (2022) indicated that the Keywords Everywhere tool assisted researchers in detecting and collecting A.I. terminologies needed to generate an A.I. vocabulary list from companies' annual reports, bridging the gap between actual narratives and expected ones.

Helium 10 has proven to be a successful tool for data harvesting and analysis, particularly when dealing with diverse data sources. In a Japanese study assessing the feasibility of expanding eBrands' global business to Amazon Japan, Helium 10 was used to collect purchasing data history and purchasing volume intelligence. JungleScoutt and Helium were the main tools used, with JungleScoutt's accuracy reported at 84.1 % and Helium's accuracy at 74 %, as mentioned by Connolly [63]. In an observational study, Niburski [39] discussed the impact of Trump's promotion of unproven COVID-19 treatments and subsequent internet trends. The method of collecting data, purchasing products, and searching volumes through Amazon was conducted using Helium, indicating that many application cases have employed Helium 10 as the primary tool for collecting, analyzing, and searching for volumes through Amazon.

The Keywords Everywhere tool was used to collect search volume for selected categories between January 2017 and March 2022. The tool's data was formatted monthly as needed, with no missing values requiring filling in. On the other hand, Helium 10: The X-ray tool has varying start dates, but all end in March 2022, necessitating pre-design to convert the data to the desired form.

Table 1 delineates the features utilized in the final dataset. The ‘Date’ column spans from January 2017 to March 2022, encompassing each month. The ‘Search Volume’ column denotes search volumes extracted from the Keywords Everywhere tool in the United States during the same period. The ‘Amazon Search Volume’ column signifies global search volumes on Amazon, extracted from the Helium 10 tool from January 2019 to March 2022.

**Table 1:** Descriptive features for the empirical data.

Feature name	Feature description	Range of values
Category	33 keywords of the various products	33
Date	month from January 2020 to March 2022	12
Search volume	search volume extracted from keywords everywhere	441400–723700
Amazon search	search volume on amazon extracted from the helium 10	115344–499383
Brand sales1-3	actual purchase for three brands	144–53038
Sales Rank1-3	sales rank for three brands	70–109759
New Price1-3	new price for three brands	40–143
List Price1-3	the list price for three brands	65–75
Review Count1-3	customers' reviews on products for three brands	1–40309
Rating1-3	products rated by customers for three brands	4.25–4.5

The ‘Category’ column encompasses 37 keywords representing various products that served as inputs for the API tools. This process resulted in a substantial number of data tables, organized as follows: six sheets per category extracted from the Keywords Everywhere tool, with only one of the six utilized for the ‘Search Volume’ feature. Simultaneously, from the Helium 10 tool, we extracted actual purchase features for five brands, classified as the highest-selling in their respective category. Each of the five brands within a category yielded three data tables, accompanied by 13 metadata entries for each brand.

It is noteworthy that the chosen five brands were classified as the highest-selling in their respective category. The next step involved specifying non-arbitrary and clustered keywords. Keywords were grouped in clusters, with a few keywords representing a cluster with similarities, such as “computer monitor” and “office chair.” This approach facilitates the analysis of categories both individually and in groups. Categories were then organized based on Amazon departments, as outlined in Table 2. Aggregating data in this manner enhances data visualization and analysis.

**Table 2:** Categories departments.

Group	Department	Categories	Group	Department	Categories
1	Shoes	running shoes nursing shoes	7	Home Security System	security cameras video doorbells
2	Home Office Furniture	computer monitor office chair	8	Arts & Crafts	epoxy resin hot glue gun paint brush macramé thread
3	Sports & Outdoor	hiking jacket tent Sleeping Bag inflatable pool board games	9	Supplements	Ashwagandha Supplements liquid chlorophyll psyllium husk
4	Gardening	planters plant growing lamps	10	Health Care Products	hearing aid elderly walker posture corrector
5	Exercise & Fitness	yoga mat Dumbbell Set treadmill Smart watch	11	Car Products	Car Phone Holder Portable Car Vacuum
6	Kitchen	bread machine Baking Mat Cooling Rack portable blender			

Table 2 shows the data grouping into departments, where 11 departments were set as follows: Shoes, Home Office Furniture, Sports & Outdoor, Gardening, Exercise & Fitness, Kitchen, Home Security System, Arts & Crafts, Supplements, Health Care Products, and Car Products.

### 3.2 Data pre-design and preprocessing

The data pre-design stage commences right after data extraction from the APIs. This stage entails converting the data obtained from the APIs into a format suitable for storage in the dataset, ensuring it is ready for analysis and experimentation.

**Fig. 2:**
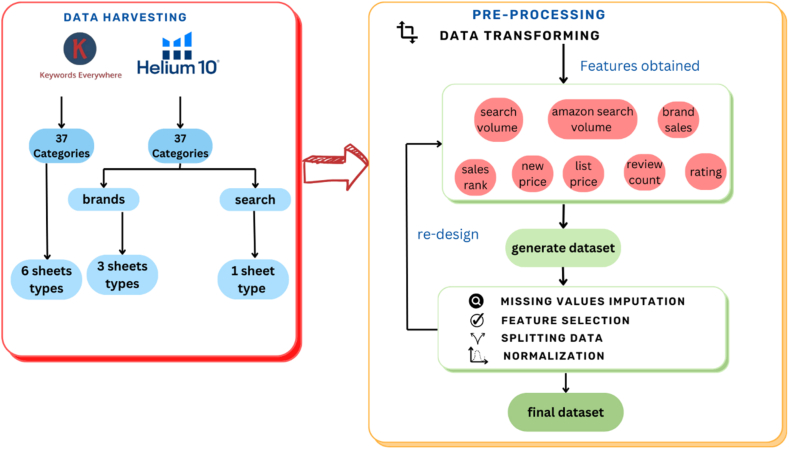
Data pre-design framework.

Fig. 2 shows, on the left-hand side, the sheets obtained from the 37 keywords harvested from the APIs, where each keyword represents a category. The Keywords Everywhere tool generated six different types of sheets for each keyword, but only one was used to get the ‘search volume’ feature.

The same 37 keywords were extracted using the Helium 10 tool, and each keyword contains three types of data: Data about searches that result in one type of sheet with the feature ‘amazon search volume’, x-ray, which was not used in this study, and data about brands that result in six types of sheets for each keyword.

The following step is represented in the right-hand side of Fig. 2, where after all the data has been harvested, it will be transformed to produce the following features: ‘search volume’ ‘amazon search volume’, ‘brand sales', ‘sales rank’, ‘new price’, ‘list price’, ‘review count’, and ‘rating’. As soon as the data is transformed, the results are combined to generate the dataset, which is then subjected to the rest of the pre-processing and data validation. The pre-design phase runs concurrently with all other phases, from pre-processing to the experimental phase.

#### 3.2.1 Data transforming

**Fig. 3:**
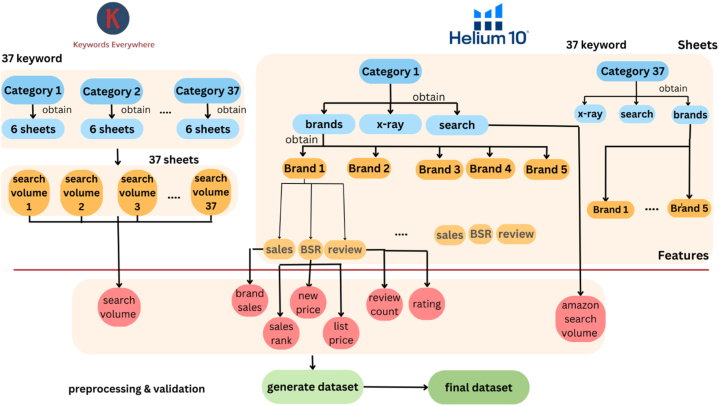
Data Pre-design Framework - feature engineering step.

In this stage, the data transformed after being harvested from the APIs, as shown in Fig. 3.

Fig. 3 illustrates that 37 categories were derived from the Keywords Everywhere tool, each consisting of 6 sheets. All sheets, except one, were disregarded, resulting in the “search volume” feature obtained from 37 sheets. The Helium 10 tool was utilized to extract the same 37 keywords, where each keyword represents a category. Each category comprises three types of data. The first type is Amazon searches, generating 37 sheets and forming the feature ‘Amazon search volume.’ The second type is x-ray sheets, which were not utilized in this study.

The last data type pertained to brands, representing data about actual purchase transactions and related information. This resulted in six types of sheets for each keyword, distributed as follows: five brands were selected for each category based on the highest sales record in a category. Each brand obtained three sheets for sales, BSR, and reviews. The ‘brand sales' feature was derived from the 37 sales sheets, while ‘review count’ and ‘rating’ features were obtained from the 37 review sheets. BSR (.) 37 sheets resulted in the features ‘sales rank,’ ‘new price,’ and ‘list price.'

Although the “search volume” was ready for storage in the dataset, other features required handling: “search volume,” “Amazon search volume,” “brand sales1-3,” “Sales Rank1-5,” “New Price1-5,” “List Price1-5,” “Review Count1-5,” and “Rating1-5.” As the data was dispersed, several iterations were necessary before preparation, including transforming the data to a monthly basis and imputation of missing values.

The data collected from the Helium 10 tool was not in the required monthly format for our study, where multiple records for the same month were taken on separate days. To convert the data to a monthly basis, the summation was computed for “Amazon search volume” and “sales” values in the same month from the same year to find the monthly value. The meanings of “new price,” “list price,” and “rating” were not specified. On the other hand, the median was computed for “sales rank” due to the diversity of data, which could introduce bias in the results if the mean was chosen instead. Finally, for “review count,” the summation was calculated, and the current month's value was subtracted from the previous month to obtain the actual review count for the current month. For example, after computing the summations, the value in June 2021 was “5628,” and in July 2021, it was “5966.” After subtraction, the final values were 355 for June 2021 and 338 for July 2021. All resulting values from these calculations were stored in the dataset, preparing it for the next step.

#### 3.2.2 Missing values imputation

Missing datasets in real-world data are a common problem for various reasons, as discussed by Guanjin Wang et al. [40]. It is rare to find a dataset that is complete and free of missing values due to the challenges and limitations faced during real-world data collection processes. Addressing and handling missing data appropriately is critical to ensure the accuracy and reliability of data analysis and modeling results. Furthermore, the presence of missing values in the dataset can impact both real-time sales monitoring and future sales forecasting. To address this issue, the initial step involved examining the dataset, with a specific focus on the “search volume” feature, which had data available starting from January 2017. This can be attributed to various factors, the most significant being the data collection period, which concluded in April 2022. However, the Helium 10 tool only retains sales data for three years, explaining the missing data in the earlier years.

Several measures were taken to mitigate the impact of missing values. Firstly, the number of brands was reduced, thereby decreasing the occurrence of missing values. Additionally, the start date of the data was adjusted, resulting in a decrease in missing values for the analyzed period. These steps aimed to improve the dataset's completeness and ensure more reliable and comprehensive results in future analyses and forecasts. Although the missing values significantly decreased, some features still required a procedure to eliminate them. Certain features were filled using the Last Observation Carried Forward (LOCF) and the Next Observation Carried Backward (NOCB) methods, depending on the location of the missing value. For “new price,” “list price,” “review count,” and “rating,” LOCF and NOCB were applied. The “sales rank” feature, however, was filled with the median over a 12-month period. These methods could not be applied to the “brand sales” feature due to its seasonality and other factors. His led us to search for another reliable method compatible with time-series data to fill the missing values in the sales. Li et al. [41] proposed a statistical learning-based approach using a regression algorithm, while Shahbazi et al. [42] suggested a regression imputation framework for hourly-based time-series data.

Due to a two-year gap between the harvested data from the Keywords Everywhere tool and the Helium 10 data, the number of data records was reduced by shifting the data's start date to January 2020 to reduce missing values. The “brand sales” feature was handled more intricately using a linear regression method, a common approach for such problems [41,42]. This involved predicting missing values and then filling in the blanks with those predictions. Two experiments were conducted using the linear regression model to impute missing values in the “brand sales” feature. The first involved applying the model to the entire dataset, while the second focused on applying it to each category separately.

In the first experiment, a regression method was applied to the entire dataset. The model was trained using available data and used to predict missing values, which were then used to fill in the blank cells. However, this led to data distortion as the values filled in for the missing data were deemed unreasonable, disrupting the natural curve and seasonality of the data.

The provided screenshot in Table 3 shows a sample of the dataset, with the filled missing values highlighted using the regression prediction results. When compared to the adjacent actual values, it is clear that the shaded cells, which represent the values filled using the regression model, exhibit extreme values. It shows brand sales in March 2020 = 55702 (predicted value) and 678 in April 2020 (actual value), which represents a high diversity. To that end, a second experiment was required to obtain reliable results.

**Table 3:** First regression imputation.

Date	category	search volume	amazon search volume
Jan 2020	179100	77914	14690
Feb 2020	186400	110201	12539
Mar 2020	201000	111449	55702
Apr 2020	361800	189765	678
May 2020	365500	230292	221

Based on the results of the first experiment, it became necessary to conduct a second experiment to address the issue of extreme values. In the following experiment, the data was partitioned into separate sections based on the categories, producing 33 distinct datasets, each corresponding to a specific category. Similarly to the first experiment, no normalization or feature selection methods were used, and this process was carried out prior to dividing the dataset into training and testing sets. Missing values in each of the 33 datasets were inputted using the regression model individually for each dataset.

In contrast to the previous experiment, the shaded values in Table 4, which represent the imputed values using the regression model, appear more reasonable. For instance, brand sales were projected to be 221 in March 2020, while the actual figures for April and May 2020 were 678 and 221, respectively. This suggests that the predicted and actual values fall within a narrow range, indicating improved accuracy. Given these positive results, the imputed values from this experiment were accepted, and the missing values in the “brand sales” feature were appropriately filled. Finally, the datasets were merged to create the final one.

**Table 4:** Second regression imputation.

Date	category	search volume	amazon search volume
Jan 2020	179100	77914	6533
Feb 2020	186400	110201	8301
Mar 2020	201000	111449	221
Apr 2020	361800	189765	678
May 2020	365500	230292	221

#### 3.2.3 Date duplication

The data has an issue of date duplication since the data contains 33 categories and each one got the same dates as the others, which is a serious problem faced us in conducting the models that needed to be solved where some algorithms refused to accept the data with this form others had an issue with performance.

The category feature was set as a unique element, using a nested for loop, where each category split the first 21 months from January 2020 to September 2021 for the training set and the remaining 6 months from October 2021 to March 2022 for the testing set. Because the data contains 27 months to predict each category, the data was split into 70 % training and 30 % testing. The timestamp was used as well in the nested loop to take each two months as input and predict the following month respectively until the end of records.

#### 3.2.4 Feature selection

According to González-Vidal et al. [34], Feature Selection involves removing irrelevant features for the task. This operation simplifies model learning by reducing data dimensions, storage requirements, and operation time, as expounded by Gupta et al. [43]. The complexity of multivariate time series data can impact prediction effectiveness. Some features are self-contained, while others exhibit correlation and cause-effect relationships. Accurate multivariate prediction requires highly correlated features while maintaining manageable time complexity. In essence, feature selection filters out paramount features and eliminates data redundancy before feeding it to the training model. Not all features in the dataset are equally important, allowing for greater explainability.

In this study, two types of feature selection were applied to choose the most relevant features among the data and enhance model performance. Two feature selection methods were used: The correlation coefficient, according to L-Dannecker [44], quantifies the degree of linear correlation between two sets of data. It is the product of the covariances of two variables and their standard deviations, essentially providing a normalized measurement of covariance with results always falling between 1 and -1. This measure, like covariance, can only reflect a linear correlation of variables and disregards other types of correlations.

According to Garate-Escamila et al. [45], the Chi-square test (CHI) arranges features by class and identifies the most important features correlated to the class label. When the observed and expected counts within two features are close, they are independent, and the Chi-Square value is smaller. The independence hypothesis is incorrect if the Chi-Square value is high. In other words, a feature with a higher Chi-Square value depends more on the response and can be chosen for model training. The primary objective of these methods is to eliminate the most unrelated features or those that may have a negative impact on the data, ensuring that features with robust relationships are retained, thereby safeguarding the accuracy of the model's predictions.

**Fig. 4:**
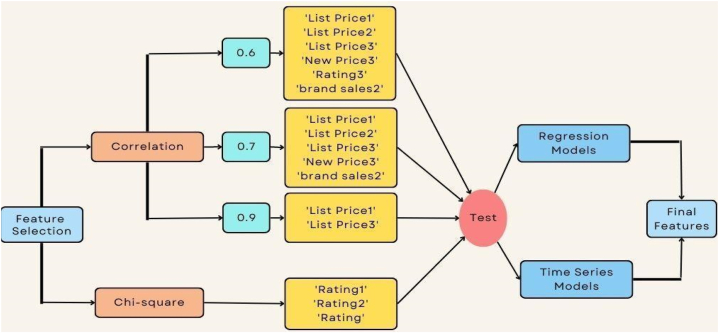
Feature selection process.

Fig. 4 demonstrates the feature selection process using two methods, “Pearson Correlation” and “chi-square” defined. The following step is to eject the self-contained features and then apply the algorithms. After eliminating self-contained features in the experiment chapter, the algorithms will be tested on the data before feature selection and on each set of features. The final set of features was chosen based on the performance of the algorithms’ experiments.

The first feature selection method used was Pearson Correlation, where the first correlation threshold was set to 0.6, which means that we want to return the features that are 60 % or more correlated with other features. Applying 0.6 thresholds on the data resulted in the following features: “List Price1”, “List Price2”, “List Price3”, “New Price3”, “Rating3”, and “Brand Sales2”. According to the Pearson Correlation method, the features obtained from this test should be excluded because they express that there is a high relationship between features that may be considered data redundancy, as discussed in Chapter 3. The second threshold = 0.7 yielded the following results: “List Price1”, “List Price2”, “List Price3”, “New Price3”, and “brand sales2”. While setting the threshold to 0.9, resulting in: “List Price1”, and “List Price3".

Furthermore, applying Chi-square on the dataset resulted in the elimination of the features “Rating1,” “Rating2,” and “Rating3".

#### 3.2.5 Splitting data into training and testing

The dataset was split into a train set that runs from January 2020 to September 2021 (21 months) and From October 2021 to March 2022 (6 months) for the test set. In other words, the training set contains 77.8 of the data and 22.2 for the testing set. The dataset in this research is characterized by date replication, where each category in the dataset carries the same dates as its peers. To simplify the problem, the dataset can be pictured as a collection of multiple datasets with the same features, dates, and periods, but grouped together in the same dataset.

Some models exhibit difficulty in prediction, as certain prediction models cannot handle date replication, leading to a deadlock. Attempts to divide each category in a dataset and apply algorithms separately to each dataset may lead to issues such as overfitting, inefficient results, and time consumption. We resolved the problem by splitting the data into training and testing sets using the proposed date replication solution method to address this.

#### 3.2.6 Normalization

Feature scaling is a method employed to normalize the range of data features. Typically, the raw data exhibits a wide variation in values, and certain algorithms may not function optimally without normalization. As many algorithms rely on calculating the distance between two points using Euclidean distance, a feature with a wide range value can dominate the distance computation. This underscores the significance of normalization, ensuring that each feature contributes approximately proportionately to the final distance. Additionally, feature scaling enhances the efficiency of gradient descent [46].

In this study, normalization was applied to generate more interpretable results from Mean Absolute Error (MAE), Mean Squared Error (MSE), and Root Mean Squared Error (RMSE) evaluation measures. The results obtained from these evaluators are initially challenging to interpret and necessitate some calculation. Since these evaluators utilize the same scale as the data being measured, they cannot be directly used to compare series with different scales. Therefore, the min-max normalization method was employed in this study.

### 3.3 Utilized machine learning models

The following are the set of ML approaches used in this study:

**XGBoost:** XGBoost, or Extreme Gradient Boosting, is a powerful machine learning model belonging to the gradient boosting algorithm family. It excels in prediction tasks, leveraging a collection of tree-boosting models. Key components include gradient boosting, regularization for preventing overfitting, tree pruning for model simplification, parallel and distributed computing for efficiency, handling missing data, and early stopping for optimizing training time.

**Random Forest:** Random Forest is a versatile machine learning model widely used for classification and Regression. It's an ensemble of decision trees, each trained independently on different subsets of data, with random feature selection to enhance diversity. Components include ensemble learning, random feature selection, voting or averaging for predictions, bagging for variance reduction, out-of-bag error estimation, variable importance insights, resilience to missing values, parallelization for scalability, hyperparameter tuning, and a trade-off between interpretability and complexity.

**Long Short-Term Memory (LSTM):** LSTM is a type of recurrent neural network (RNN) designed for sequential data. It excels in tasks with lags between events or trends. Components include sequential data handling, capturing long-term dependencies, gating mechanisms for information flow control, cell state for storing information, addressing the vanishing gradient problem, peephole connections for enhanced dependency capture, bidirectional processing for future context, stacked layers for model depth, dropout for regularization, and hyperparameter tuning.

**Linear Regression**: Linear Regression models the linear relationship between dependent and independent variables. Components include a linear relationship assumption, regression coefficients for feature impact, intercept term, the least squares method for minimizing residuals, assumptions for accuracy, scalability for large datasets, and a trade-off between simplicity and capturing complex relationships.

**K-Nearest Neighbors (KNN):** KNN is an analogy-based learning algorithm predicting based on similarities between training and testing sets. Components include the number of neighbors (K) as a crucial hyperparameter, distance metrics for similarity measurement, saving and searching the entire training dataset for predictions, flexible decision boundaries, hyperparameter tuning for optimal K value, limitations in scalability due to computational costs, and the importance of feature scaling and handling missing data considerations.

### 3.4 Evaluation measures

In this research, R2, MAE, MSE, and RMSE evaluation measures were applied to the data. The coefficient of determination, denoted as R2, serves as a pivotal metric in assessing the models' performance in other words R^2^ used for indicating the models' performance. It expresses how well the models capture the variance in the data. On the other hand, the trio of Mean Absolute Error (MAE), Mean Squared Error (MSE), and Root Mean Squared Error (RMSE) stand as pivotal tools for gauging the models' predictive accuracy and error. These measures collectively provide insight into how closely the models' predictions align with the actual observed values, offering a comprehensive evaluation of the models' performance.

## 4 Data analysis and visualization

It should be noted that the figures in this section have been reduced in size to improve readability. The full-sized versions are included in the appendix section.

### 4.1 Amazon search volume

**Fig. 5:**
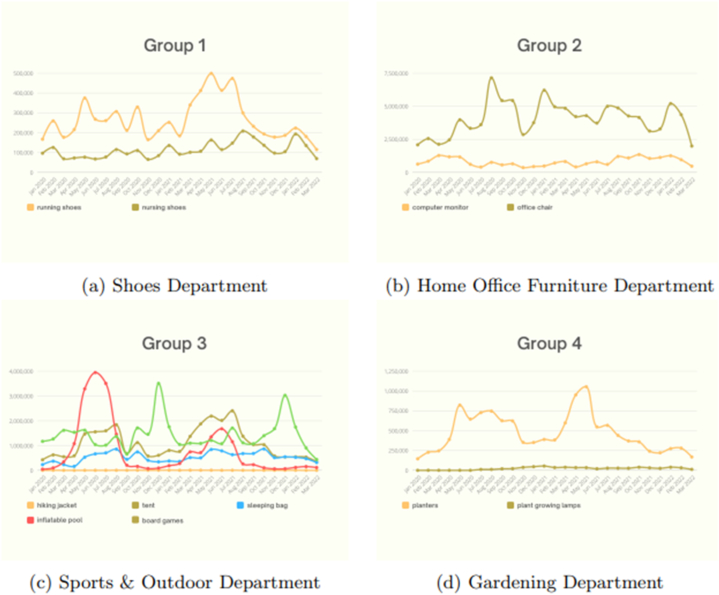
Amazon search volume (groups 1–4).

**Fig. 6:**
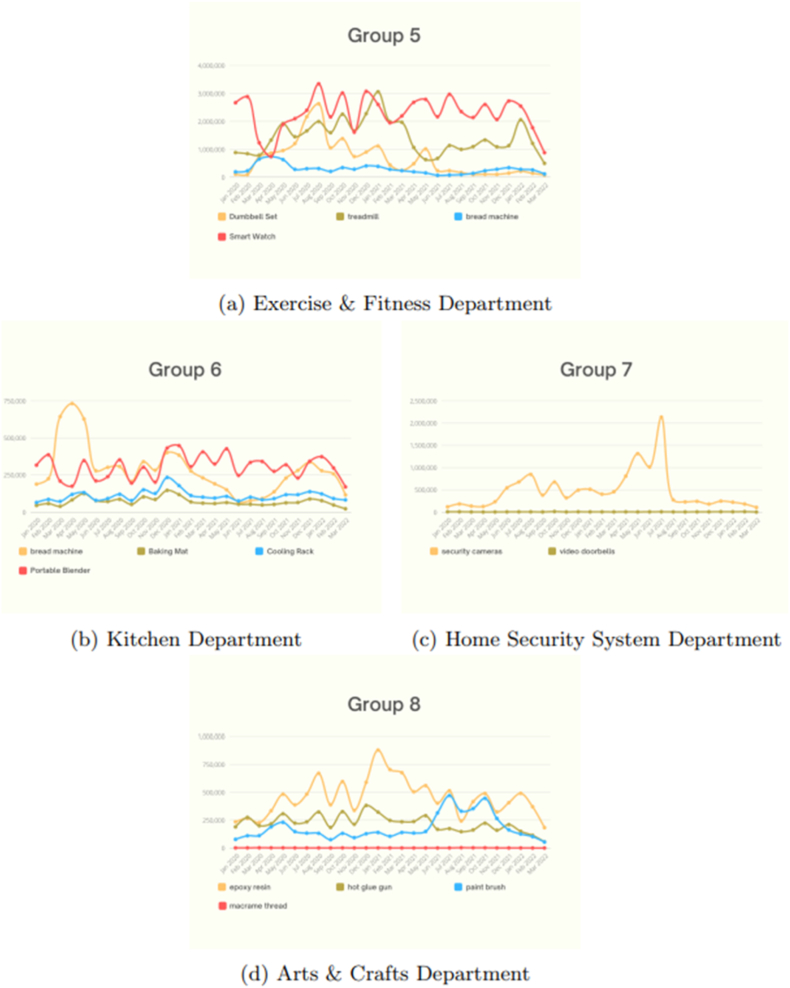
Amazon search volume (groups 5–8).

In this section, the Amazon search volume for categories is grouped by departments in order to observe the behavior of various categories within the same group. Each group has similarities in that they may share functionality, purpose, or seasonality since they might be summer or winter products. The categories similarly fluctuate in a similar way, as shown in Fig. 5 (a - d). Even if they did not coincide in the same data point, due to the differentiation of the data scale, they experienced roughly the same increase and drop over the same period. However, some categories in a specific group may not visualize well, as they do not show the trends clearly, where the scale of the rest of the categories exceeds that category scale in a particular group. Fig. 6 (a – d), for example, clearly demonstrate this.

### 4.2 Search and sales relation

**Fig. 7:**
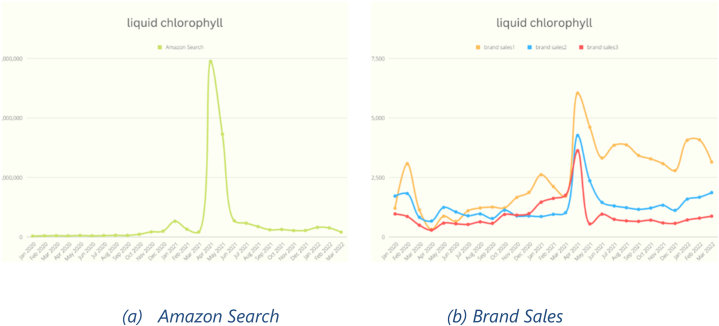
Liquid Chlorophyll Search vs. Sales.

**Fig. 8:**
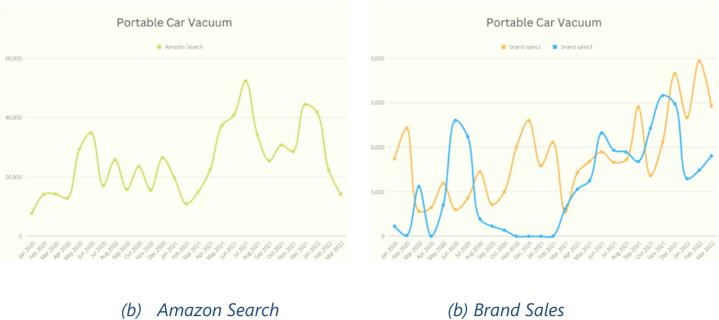
Portable Car Vacuum Search vs. Sales.

As this research assumes a connection between search volume and actual purchase, the investigation will delve into nurturing this relationship to determine its true nature and explore why it may appear disconnected in certain cases. While the data for “liquid chlorophyll” and “portable car vacuum” Fig. 7, Fig. 8 show a clear and evident correlation between search volume (Fig. 7, Fig. 8) and actual purchase (Fig. 7, Fig. 8), some other categories exhibit dissimilarities. These differences can be attributed to the nature of the data, where numerous brands sell products within the same category, but only a few were included in this study. Consequently, the actual purchase data becomes sparse among all the brands, leading to potential discrepancies in the observed trends.

To obtain a more comprehensive picture and align the trends, it would require collecting data from all brands that sell products within the same category. However, this process can be challenging and time-consuming due to the sheer number of brands and products involved. Only then, with a more complete dataset encompassing all brands, could a clearer relationship between search volume and actual purchase be established.

### 4.3 COVID-19 influence

As mentioned earlier, various factors such as seasons, holidays, and major events like the COVID-19 pandemic can significantly influence data trends. This section will focus on understanding the impact of COVID-19 on data trends using a sample of nine categories extracted from the Keywords Everywhere tool. The data spans from January 2017 to March 2022, as the Helium 10 tool does not provide sufficient data to observe the influence of COVID-19 on trends.

The data related to COVID-19 influence is categorized into three major periods. The first period, termed “pre-COVID-19,” covers January 2017 to December 2019. The second period, “COVID-19,” spans from January 2020 to June 2021, with March 2020 considered as the peak of COVID-19 influence based on the available data. This peak coincides with the implementation of lockdown procedures, which likely caused significant changes in individuals' behavior as they adapted to new needs and circumstances. The final period, “post-COVID-19,” encompasses July 2021 to March 2022.

Throughout this analysis, this research aims to identify any notable shifts or patterns in the data trends caused by the COVID-19 pandemic during these specified periods.

**Fig. 9:**
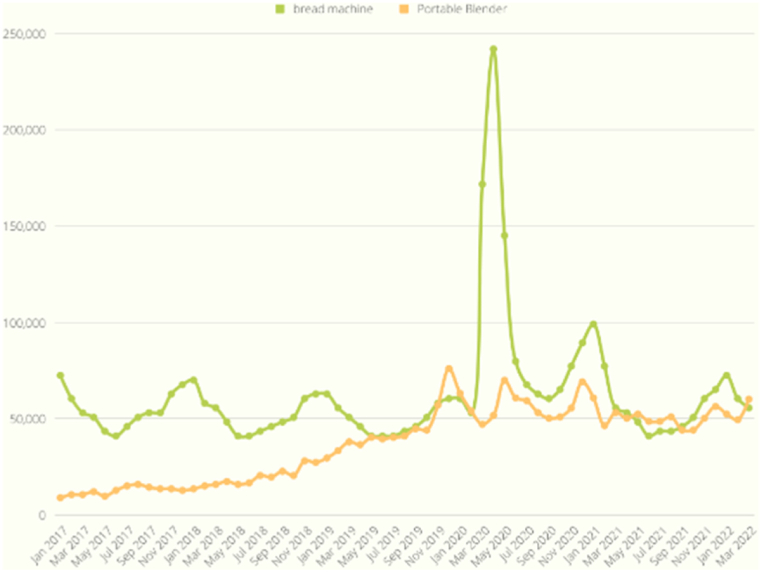
Bread machine and portable blender google search volume.

**Fig. 10:**
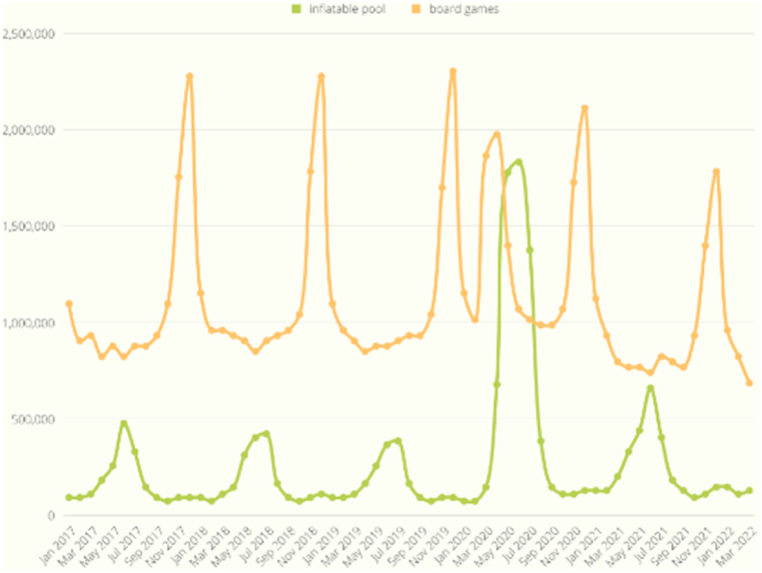
Inflatable pool and board games google search volume.

**Fig. 11:**
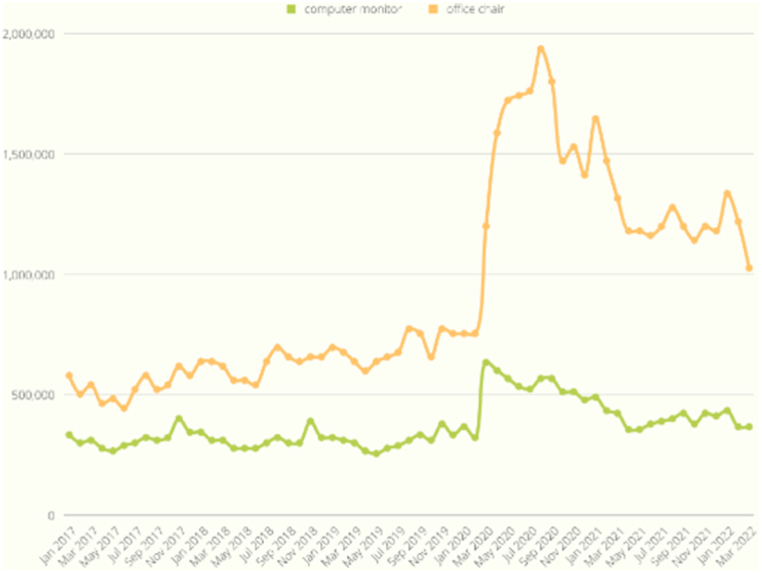
Computer monitor and office chair google search volume.

**Fig. 12:**
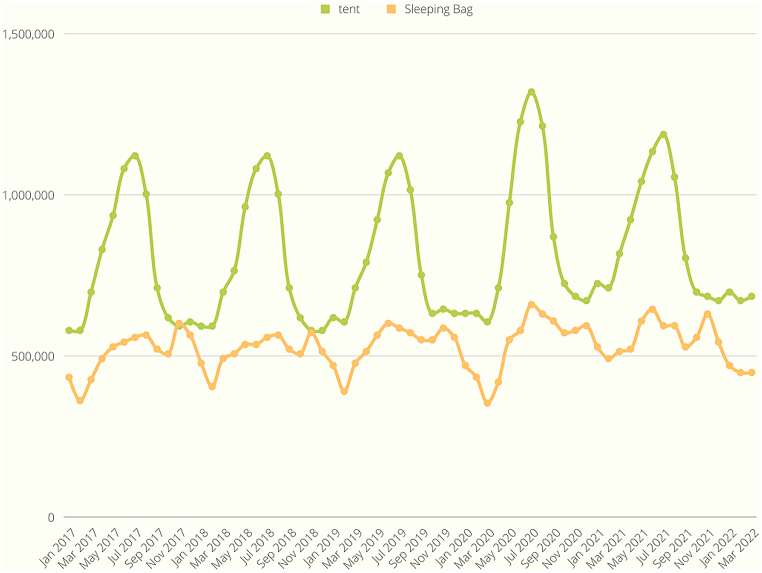
Tent and sleeping bag google search volume.

**Fig. 13:**
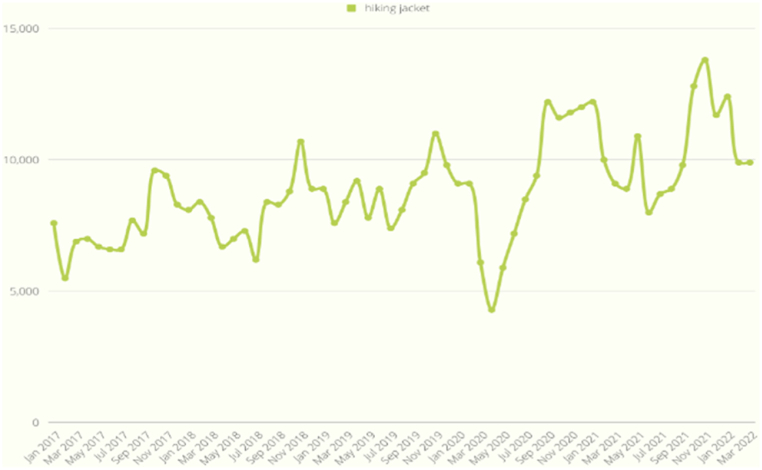
Hiking jacket google search volume.

As shown in Fig. 9, the search volume exhibited a synchronized increase with the onset of COVID-19, evident in the case of the bread machine, which reached its peak in March 2020. This trend similarly applied to the inflatable pool in Fig. 10 and the office chair in Fig. 11. On the other hand, Fig. 12 displays a slight increase in search volume for the tent and sleeping bags during the COVID-19 period, but it did not disrupt the regular data seasonality. Conversely, Fig. 13 illustrates a decline in search volume for the hiking jacket during the same COVID-19 period.

The data clearly demonstrates that certain categories, such as the office chair, have adopted a new trend in the post-COVID-19 period, often referred to as the “New Normal.” This transformation can be attributed to the impact of COVID-19 on individual behaviour, as there was a notable shift towards working from home, leading to an increased demand for office chairs. In conclusion, the analysis of COVID-19's impact on data trends reveals a significant influence on the purchase curve. The distortions caused by the pandemic are likely to have repercussions on predictive models, as the curve has undergone notable changes during this period.

## 5 Experimental results

The time series data, as previously stated, spans from January 2020 to March 2022, whereas the training data spans from January 2020 to September 2021, with the remaining data set for testing until March 2022. The data encompasses 33 categories and 20 additional features with the ultimate goal of predicting “Amazon search volume".

This section focuses on experimenting with time series data using machine learning models, namely XGBoost, RF, LSTM, Linear Regression, and KNN. For the nearest neighbor's parameter in K-Nearest Neighbors, six distinct configurations were chosen (KNN). These parameters were 5, 7, 8, 10, 12, and 15. This range was determined based on practical observations, as lowering the value below 5 results in a deterioration of all quality indexes, while raising the value above 15 results in similar unfavorable outcomes.

The objective is to use evaluation measures to assess their performance in various settings and examine their behavior where the performance in those experiments was evaluated using R2, MAE, MSE, and RMSE. Moreover, in this experiment, research was carried out using the min-max normalization method. For the feature selection, two methods were used, where each experiment varied from another with the used feature selection method as summarized in Table 5. The correlation feature selection was set to three different values: 0.6, 0.7, and 0.9, where 0.9 is our ceiling because it only excludes two features, whereas 0.6 excludes six features, and lowering the value causes a lower performance, as observed in the preliminary experiments.

**Table 5:** Feature selection method used in each experiment.

Feature Selection Method	Experiment used (Type if any)
No Feature Selection	1, 2
Pearson Correlation	1(all), 3(0.6), 4(0.7), 5(0.9)
Chi-Square	1, 6

### 5.1 Experiment 1

The experiment was carried out on unnormalized data in order to assess the performance of the models. This method allowed for a comparison of results obtained prior to normalization and those obtained after normalization. To monitor performance, the models (XGBoost, RF, Linear Regression, and KNN) were applied before and after feature selection, which included the two types of feature selection.

Due to the absence of data scaling in this experiment, only R2 scores were computed. This decision was made because the utilization of other evaluation measures, as described in the methodology, would have been difficult to interpret if the data had not been scaled.

**Results**: The first experiment series performance of XGBoost, RF, Linear Regression, and KNN models obtained using R2 measure is shown in Table 6. Random Forest has low overall performance results, in addition to performance instability. For example, we ran random forests ten times with a correlation of 0.7 and got different results each time. The lowest performance was 71.65, and the highest was 73.7, indicating a variance of about 2 points. On the other hand, XGB, L.R., and KNN have a consistent performance; we ran each one about five times, and the results were consistent each time. XGB outperformed with correlations of 0.7 and 0.9 by 76.70 R2 and performed worse with correlations of 0.6 by R2 = 74.80. The L.R. model performed well with nuance, but the Chi-square feature selection produced the best L.R. by R2 = 80.84. The KNN model may not appear to be very promising based on the first result of R2 72.88 with KNN = 5 and no feature selection applied, but in this experiment, KNN performed well with an R2 score of 81.28, KNN = 15 with feature selection correlations of 0.6, 0.7, and 0.9. In general, KNN improves as the number of nearest neighbors increases, but it had the lowest R2 score results with chi-square.

**Table 6:** Results of experiment 1 - regression algorithms evaluation after normalization.

Algorithm	no-normalization	Corr 0.6	Corr 0.7	Corr 0.9	Chi-Square
XGB Regressor	75.66	74.80	76.70	76.70	74.84
Random Forest Regressor	71.83	71.33	73.11	71.96	72.25
LSTM	–	–	–	–	–
Linear Regression	80.33	80.33	80.36	80.36	80.84
KNN Regressor = 5	72.88	73.11	73.11	73.10	74.61
KNN Regressor = 7	77.80	77.81	77.81	77.81	74.57
KNN Regressor = 8	78.23	78.21	78.21	78.21	75.69
KNN Regressor = 10	77.56	77.61	77.61	77.61	75.89
KNN Regressor = 12	79.17	79.18	79.18	79.18	75.22
KNN Regressor = 15	81.18	81.28	81.28	81.28	78.01

### 5.2 Experiment 2

In addition to the benefits of improving speed and convergence with feature scaling via gradient descent, the wide range of values in our dataset may distort model performance if not properly scaled. Without data scaling, interpreting the results of evaluation measures such as Mean Absolute Error (MAE), Mean Squared Error (MSE), and Root Mean Squared Error (RMSE) could be difficult. To address these concerns, the data in this experiment was scaled using min-max normalization. The goal of this approach was to keep the results consistent and equivalent. Furthermore, no feature selection technique was used in this experiment to ensure a thorough evaluation of all available features.

**Results**: The results of this experiment are succinctly summarized in Table 7-(Normalization column), which reveals that the models XGBoost, RF, Linear Regression, and KNN generate good forecasts, however, they all have the same MSE score: 0.003, but they differ by minor differences in MAE and RMSE. Where XGBoost had the best MAE result with a 0.026 score, followed by R.F. and Linear Regression, both of which had a 0.027 MSE, KNN with 12 and 15 nearest neighbors produced the lowest MSE result with a 0.032 score. In terms of RMSE Linear Regression produced the best result with a 0.051 score, followed by KNN with 5 and 7 nearest neighbors by a score of 0.055 and the R.F. hit the minimal RMSE with a 0.059 score. On the other hand, Linear Regression produced the best R2-score of 80.33, followed by KNN with 7 nearest neighbors by 80.02 R2-score, and R.F. produced the lowest R2 with a 72.81 score. Linear Regression produced the best results, with the highest R2, MSE, and RMSE respectively of 80.33, 0.003, and 0.051 scores, and second place in MAE with a 0.027 score. LSTM did not appear to fit the data well, as it has the poorest results, particularly with R2 = −8839.37, besides it hit the lowest results in MAE, MSE, and RMSE respectively of 0.137, 0.022, and 0.148 scores.

**Table 7:** Results of experiments 2–4 - regression algorithms evaluation after normalization.

Algorithm	Metric	Normalized	Corr 0.6	Corr 0.7	Corr 0.9	Chi-Square
**XGBoost**	R2	73.956	74.705	75.072	76.241	73.341
	MAE	0.026	0.026	0.025	0.026	0.026
	MSE	0.003	0.003	0.003	0.003	0.003
	RMSE	0.058	0.058	0.057	0.056	0.058
**Random Forest**	R2	72.806	74.120	71.704	72.457	72.620
	MAE	0.027	0.027	0.026	0.027	0.027
	MSE	0.003	0.004	0.003	0.004	0.004
	RMSE	0.059	0.059	0.060	0.061	0.060
**LSTM**	R2	−8839.370	−45	−61.398	−1917.632	−712524.504
	MAE	0.137	0.160	0.124	0.487	0.974
	MSE	0.022	0.031	0.032	0.255	0.960
	RMSE	0.148	0.177	0.181	0.505	0.980
**Linear Regression**	R2	80.333	80.331	80.356	80.397	80.837
	MAE	0.027	0.027	0.027	0.027	0.026
	MSE	0.003	0.003	0.003	0.003	0.002
	RMSE	0.051	0.051	0.051	0.051	0.050
**KNN** = **5**	R2	79.735	80.570	80.762	79.783	79.143
	MAE	0.029	0.028	0.027	0.029	0.030
	MSE	0.003	0.003	0.003	0.003	0.003
	RMSE	0.055	0.055	0.054	0.055	0.052
**KNN** = **7**	R2	80.016	79.577	79.036	79.819	76.835
	MAE	0.028	0.028	0.029	0.028	0.032
	MSE	0.003	0.003	0.003	0.003	0.003
	RMSE	0.055	0.056	0.057	0.056	0.053
**KNN** = **8**	R2	79.225	79.403	79.468	79.199	73.912
	MAE	0.030	0.029	0.029	0.030	0.033
	MSE	0.003	0.003	0.003	0.003	0.003
	RMSE	0.058	0.057	0.057	0.058	0.056
**KNN** = **10**	R2	79.376	80.248	79.629	80.248	69.079
	MAE	0.030	0.030	0.030	0.030	0.033
	MSE	0.003	0.003	0.003	0.003	0.003
	RMSE	0.057	0.055	0.056	0.055	0.058
**KNN** = **12**	R2	77.704	78.420	78.155	77.905	66.600
	MAE	0.032	0.031	0.031	0.031	0.032
	MSE	0.003	0.003	0.003	0.003	0.003
	RMSE	0.058	0.057	0.057	0.057	0.058
**KNN** = **15**	R2	78.711	79.050	78.547	78.699	61.988
	MAE	0.032	0.031	0.032	0.032	0.035
	MSE	0.003	0.003	0.003	0.003	0.003
	RMSE	0.056	0.055	0.056	0.056	0.061

### 5.3 Experiment 3

Given that some features operate independently while others exhibit correlations and interdependencies, the complexities of multivariate time series data can have a significant impact on prediction accuracy. Accurate multivariate predictions necessitate the identification of highly correlated features while managing time complexity effectively. The Correlation feature selection method was used in these experiments to quantify the level of linear correlation between two sets of data, with correlation thresholds of 0.6, 0.7, and 0.9. The data were scaled using Min-max normalization across all subsequent experiments to ensure a fair comparison of results.

**Results:**Table 7 (0.6, 0.7, 0.9 columns) summarizes the results of these predictions at correlation thresholds of 0.6, 0.7, and 0.9. The KNN model with 5 nearest neighbors performed the best, with an R2 score of 80.762 at a correlation threshold of 0.7, followed by an R2 score of 80.570 for KNN = 5 at a correlation of 0.7. The worst performance after LSTM was seen with R.F., which achieved an R2 score of 71.704 at a correlation threshold of 0.7.

The first feature selection experiment produced satisfactory results, indicating that it has the potential to improve performance in subsequent experiments. Even if the same level of performance is maintained, feature exclusion is considered advantageous because it simplifies model learning and reduces the time required for model operations. As a result, reducing the number of features is deemed a positive outcome.

### 5.4 Experiment 4

Upon applying the Chi-Square method to the time series data, the features “Rat-ing1,” “Rating2,” and “Rating3” were identified for removal. Moreover, the features excluded by this method aren't always the same as those excluded by the Pearson Correlation method, though there may be some overlap with the “Rat-ing3” feature, which was also excluded when the Correlation threshold was set to 0.6.

**Results**: Table 7 (Chi-Square column) summarizes the results of the models' performance. Where the data were obtained after conducting the Chi-Square method, which resulted in excluding 3 features. The model's performance varied up to 0.004 with MAE and MSE, and up to 0.006 with RMSE when compared to the previous experiments: 2,4,5, and 6. R2 decreased by approximately 11° with KNN = 10, 12° with KNN = 12, and 17° with KNN = 17, while the remaining models decreased by up to 5°. The best model performance in this experiment was obtained in L.R. with R2 = 80.84, MAE = 0.026, MSE = 0.002, and RMSE = 0.050 and although L.R. in this experiment was better than the previous ones, the models in the previous experiments performed better overall.

Because of the small number of excluded features with these methods, it was found that experiment with a 0.9 threshold correlation is the most relative to this experiment.

### 5.5 Hyperparameter optimization experiments

Earlier trials used base modules with default parameter settings. In order to improve performance, a new set of experiments was carried out, with hyperparameter optimization using grid search as the chosen optimization method. Extensive analyses were performed on four different machine learning models in the hyperparameter optimization experiments: XGBoost, Random Forest, KNN (K-Nearest Neighbors), and Linear Regression. These models were fine-tuned systematically to identify optimal hyperparameters that improve their predictive capabilities. Correlation thresholds of 0.6, 0.7, and 0.9 were applied uniformly across all models to ensure effective feature selection and model robustness. These correlation thresholds aided in the filtering of relevant features, resulting in a more focused set for training and improved model interpretability. Furthermore, the Minmax Scaler normalization method was used to standardize the training process and facilitate fair comparisons. The hyperparameter optimization experiments aims to uncover the most effective configurations for each model by combining these techniques, promoting accurate predictions and robust generalization to new data.

Table 8 demonstrates the hyperparameter settings for each model. The table also shows certain possible values for each hyperparameter where the bold values are the optimal ones. The hyperparameter optimization process has been cross-validated using k-fold value = 5.

**Table 8:** Hyperparameter settings.

Hyperparameter	Description	*Examined Values (Optimal values are in bold format)*
**Random Forest Tree (RFT)**		
*max_depth*	the longest path between the root and the leaf of a tree.	{10, 20, 30, 40, 50,**100**}
*min_sample_split*	the minimum number of observations that are required in any given node in order to split it.	{2, **5**, 10}
*max_features*	features to consider when observing for the best split.	{“**auto**”, “sqrt”}
*Bootstrap*	uses replacement sampling in a random sample of a dataset	{**True**, False}
*min_samples_leaf*	the minimum number of samples exist in the leaf node after splitting a certain node.	{1, 2, **4**}
*n_estimators*	specifies the number of trees in the forest of the model.	{200, 400, 600, 800, **1000**}
**Linear Regression (L.R.)**		
*fit_intercept*	indicates whether the intercept for this model should be calculated.	{‘True’, ‘**False’**}
*n_jobs*	the number of jobs that will run in parallel for certain operations.	{‘**None’**}
*copy_X*	specifies whether the input data should be copied before fitting.	{‘**True’**, ‘False’}
**XGB Regressor**		
n_estimators	controls the number of trees	{**50,** 100, 150}
max_depth	The maximum depth of each tree	{**3**, 7, 5}
*learning_rate*	the step size at each iteration as the loss function is minimized.	{0.01, **0.1**, 0.2}
**KNN**		
n_neighbors	The number of neighbors (k)	{5, 7, 9, 10, **12**, 15}
*weights*	determines how much weight each neighbor receives.	{'uniform’, **‘distance'**}
*P*	denotes the Minkowski distance metric's power parameter.	{**1**,2}

**Results**: Table 9 illustrates the outcomes derived from the Hyperparameter Optimization Experiments conducted on all selected models (XGBoost, Random Forest, Linear Regression, and KNN). These experiments were carried out with varying correlation coefficients of 0.6, 0.7, and 0.9, using the same evaluation metrics as in previous experiments, namely MAE, MSE, RMSE, and R2. It is noteworthy that all models exhibited improvements, and the hierarchy of model preferences underwent alterations. Linear Regression demonstrated superior performance, particularly at a correlation coefficient of 0.9, achieving R2 = 90.688, MAE = 0.038, MSE = 0.003, and RMSE = 0.057. In contrast, KNN performed best at a correlation coefficient of 0.7, achieving R2 = 85.129, MAE = 0.045, MSE = 0.005, and RMSE = 0.068, all of which represent significant improvements over previous experiments. For XGBoost, the optimal outcomes materialized at a correlation coefficient of 0.9, yielding R2 = 85.89, MAE = 0.042, MSE = 0.004, and RMSE = 0.062. Random Forest's performance, on the other hand, improved significantly, competing with the other models, with peak metrics at a correlation coefficient of 0.6, R2 = 84.854, MAE = 0.041, MSE = 0.004, and RMSE = 0.066.

**Table 9:** Results of hyperparameter optimization experiment.

Algorithm	Metric	Corr. 0.6	Corr. 0.7	Corr. 0.9
**XGB Regressor**	R2	84.704	84.704	85.89
	MAE	0.042	0.042	0.042
	MSE	0.004	0.004	0.004
	RMSE	0.063	0.063	0.062
**Random Forest**	R2	84.854	84.516	83.820
	MAE	0.041	0.040	0.041
	MSE	0.004	0.004	0.005
	RMSE	0.066	0.066	0.068
**Linear Regression**	R2	89.805	89.805	90.688
	MAE	0.031	0.039	0.038
	MSE	0.003	0.003	0.003
	RMSE	0.057	0.058	0.057
**KNN Regressor**	R2	85.015	85.129	82.503
	MAE	0.044	0.045	0.048
	MSE	0.005	0.005	0.005
	RMSE	0.068	0.068	0.071

## 6 Discussion

The current digital era is witnessing an unprecedented surge in information growth, driving a continual need for e-commerce solutions that align with the fast-paced nature of contemporary life. E-commerce, geared towards replacing traditional trading processes, has become a pivotal domain for businesses seeking to showcase their products or services across diverse industries. As a result, conventional enterprises are incorporating e-commerce into their operations to stay competitive in the evolving landscape.

The rapid expansion of e-commerce is fueled by the growing preference for online shopping, capturing the attention of retailers and decision-makers in both business and I.T. sectors. Accurate demand predictions are essential for effective decision-making in this dynamic environment. Customer demand, derived from search and sales volume for specific categories, forms the basis for enhanced predictions benefiting both decision-makers and individuals in the retail business. External factors such as occasions, sales events, and unexpected occurrences significantly influence consumer behavior. Occasions like holidays, Mother's Day, and major sports events impact purchasing behavior, creating substantial opportunities and challenges for retailers. Sales events like "Singles' Day,” Black Friday, and Cyber Monday have become global phenomena, influencing consumer spending patterns. Unforeseen events, such as the COVID-19 pandemic, have led to a notable shift towards e-commerce, underscoring the need for accurate sales forecasting in the face of uncertainties.

To address these challenges, retailers require precise sales demand information to make informed decisions. Accurate sales forecasting is critical for cost reduction, profit maximization, and inventory management. Insights into customer behavior, derived from search volume and historical purchase data, provide retailers with a clear vision of market trends to guide business development and achieve desired returns on investment.

This research focuses on the real-world problem faced by retailers and decision-makers, proposing an application that predicts trending products and optimal selling times. The study advocates analyzing market trends by combining individual search volume with actual sales data across diverse categories. It emphasizes the importance of a comparative study between datasets obtained from reliable tools, such as Keywords Everywhere and Helium10, using data mining methods for experimentation.

The novelty of the research lies in the diverse datasets obtained from various APIs, contributing significantly to understanding the relationship between sales volume, search volume, and other influencing factors. On a practical level, the research empowers retailers to survive and thrive by enabling accurate demand prediction, strategic stock management, and efficient supply chain planning. It emphasizes the importance of setting competitive prices, offering targeted discounts, and executing effective promotional campaigns based on insights into future demand.

The research also highlights the significance of comparing different machine learning models to evaluate performance. This comparative analysis assists in determining which models should be further considered based on their ability to outperform others. The proposed time-aware forecasting framework integrates knowledge of customer behavior at distinct time scales, adding a valuable dimension to the understanding of market dynamics in the context of e-commerce. In future work, we will be including a broader range of data over a longer period and increasing the number of keywords. Furthermore, experimenting with hybrid models that combine traditional statistical methods with advanced machine learning techniques has the potential to leverage the strengths of both approaches for more accurate predictions.

## 7 Conclusion

The passionate interest in e-commerce creates an insatiable demand for solutions that offer insights and predictions about coveted products and the best times to introduce them to the market. Businesses that are perpetually grappling with the conundrum of selecting the perfect product at precisely the right time find the key to unlocking a treasure trove of data-driven decision-making in precise demand forecasting.

Within this multifaceted landscape, data mining plays a critical role, as a slew of algorithms, such as XGBoost, have proven their mettle in providing precise demand forecasts. Furthermore, the temporal dimension encapsulated within time series data is an essential cog in the machinery of demand and sales forecasting, accelerating product delivery and providing retailers with the foresight required to offer their products to the market at precisely the right time [31].

To that end, this research is fundamentally intended to probe into the complex framework of market dynamics by combining individual search volumes with actual sales data from a variety of product categories. Furthermore, this study presents a thorough comparison of datasets obtained from two distinct sources: Keywords Everywhere and Helium10. A variety of models, including XGBoost, Linear Regression, Random Forest, Long-Short-Term Memory (LSTM), and K-Nearest Neighbor (KNN), were used for predicting market trends. Further, Hyperparameter optimization was carried out to enhance model performances across the board. Linear Regression consistently outperforms other models, especially at higher correlation thresholds. The presented results provide valuable insights into the effectiveness of different models and their configurations for predicting Amazon search volume based on the given time series data.

## Data availability statement

Data will be made available on request.

## Additional information

No additional information is available for this paper.

## CRediT authorship contribution statement

**Shahed Abdullhadi:** Writing - review & editing, Writing - original draft, Visualization, Validation, Software, Resources, Methodology, Investigation, Funding acquisition, Formal analysis, Data curation, Conceptualization. **Dana A. Al-Qudah:** Writing - review & editing, Validation, Supervision, Project administration. **Bilal Abu-Salih:** Writing - review & editing, Validation, Supervision, Project administration.

## Declaration of competing interest

The authors declare the following financial interests/personal relationships which may be considered as potential competing interests:The corresponding author serves as a section editor at Heliyon. If there are other authors, they declare that they have no known competing financial interests or personal relationships that could have appeared to influence the work reported in this paper.

## References

[1] Karine H.A.J.I. (2021). E-commerce development in rural and remote areas of BRICS countries. J. Integr. Agric..

[2] Bicevskis J., Nikiforova A., Karnitis G., Oditis I., Bicevska Z. (2021, September). 2021 16th Conference on Computer Science and Intelligence Systems (FedCSIS).

[3] Guven H. (2020). Agile Business Leadership Methods for Industry 4.0.

[4] Xiao J., Wu Y., Xie K., Hu Q. (2019). Managing the e-commerce disruption with IT-based innovations: insights from strategic renewal perspectives. Information \& Management.

[5] (2023). Holiday Retail Sales in the United States 2000-2023 | Statista.

[6] Richter F. (2023). https://www.statista.com/chart/11979/holiday-season-retail-sales/.

[7] Liberto D. (2023). https://www.investopedia.com/terms/s/singles-day.asp.

[8] Hampson L. (2019). Singles awareness day 2019: what is it and why celebrate the antidote to valentine's day? | London Evening standard. Evening Standard.

[9] A look at Alibaba's “Double 11” shopping day, the world's largest online retail event. Reuters. https://www.reuters.com/graphics/SINGLES-DAY-ALIBABA/0100B30E24T/index.html ".

[10] Shopify merchants set new Black Friday Cyber Monday record with \$7.5 billion in sales (2022). Shopify press room.

[11] Kulshrestha S., Saini M.L. (2020, December). 2020 5th IEEE International Conference on Recent Advances and Innovations in Engineering (ICRAIE).

[12] Lunn P.E.T.E.R.D. (2012). Telecommunications consumers: a behavioral eco- nomic analysis. J. Consum. Aff..

[13] Keywordseverywhere.com (2022). Browser add-on to see Google search vol- ume everywhere. https://keywordseverywhere.com.

[14] helium10.com (2022). Software for amazon FBA Sellers & Walmart Sellers. Helium.

[15] Cheriyan S., Ibrahim S., Mohanan S., Treesa S. (2018, August). 2018 Interna- Tional Conference on Computing, Electronics & Communications Engineer- Ing (iCCECE).

[16] Wang C., Hsu C.Y. (2020, May). 2020 5th IEEE International Con- Ference on Big Data Analytics (ICBDA).

[17] Pereira M.M., de Oliveira D.L., Santos P.P.P., Frazzon E.M. (2018). Predictive and adaptive management approach for omnichannel retailing supply chains. IFAC-PapersOnLine.

[18] Palkar A., Deshpande M., Kalekar S., Jaswal S. (2020, July). 2020 International Conference on Electronics and Sustainable Communication Sys- Tems (ICESC).

[19] Wu P., Chen Y. (2021). Product Demand Forecasting in Ecommerce Based on Nonlinear Autoregressive Neural Network.

[20] Chen C., Liu Z., Zhou J., Li X., Qi Y., Jiao Y., Zhong X. (2019, April). Pacific- Asia Conference on Knowledge Discovery and Data Mining.

[21] Jain A., Karthikeyan V., Sahana B., Shambhavi B.R., Sindhu K., Balaji S. (2020, November). 2020 IEEE International Conference for Innovation in Technology (IN- OCON).

[22] Abbasimehr H., Shabani M., Yousefi M. (2020). An optimized model using LSTM network for demand forecasting. Comput. Ind. Eng..

[23] Qi Y., Li C., Deng H., Cai M., Qi Y., Deng Y. (2019, November). Proceedings of the 28th ACM International Conference on Information and Knowledge Management.

[24] Chen L., Wang F. (2013). Preference-based clustering reviews for augmenting e-commerce recommendation. Knowl. Base Syst..

[25] Ajibade S.S., Adediran A. (2016). An overview of big data visualization techniques in data mining. International Journal of Computer Science and Information Technology Research.

[26] Bowen T., Zhe Z., Yulin Z. (2020, June). 2020 IEEE International Conference on Artificial Intelligence and Computer Applications (ICAICA).

[27] Zhang C., Tian Y.X., Fan Z.P. (2022). Forecasting sales using online review and search engine data: a method based on PCA–DSFOA–BPNN. Int. J. Forecast..

[28] Wachter P., Widmer T., Klein A. (2019, September). 2019 Federated Conference on Computer Science and Information Systems (FedCSIS).

[29] Sohrabpour V., Oghazi P., Toorajipour R., Nazarpour A. (2021). Export sales forecasting using artificial intelligence. Technol. Forecast. Soc. Change.

[30] Mukherjee S., Shankar D., Ghosh A., Tathawadekar N., Kompalli P., Sarawagi S., Chaudhury K. (2018).

[31] Ali R.F., Muneer A., Almaghthawi A., Alghamdi A., Fati S.M., Ghaleb E.A.A. (2023). BMSP-ML: big mart sales prediction using different machine learning techniques. Int. J. Artif. Intell..

[32] Pernambuco B.S.G., Steffens C.R., Pereira J.R., Werhli A.V., Azzolin R.Z., Estrada E.D.S.D. (2019, October). 2019 Latin American Robotics Symposium (LARS), 2019 Brazilian Symposium on Robotics (SBR) and 2019 Workshop on Robotics in Education (WRE).

[33] Kothari C. (2017). New Age International.

[34] Gonzalez-Vidal A., Jimenez F., Gomez-Skarmeta A.F. (2019). A methodology for energy multivariate time series forecasting in smart build- ings based on feature selection. Energy Build..

[35] Bandara K., Shi P., Bergmeir C., Hewamalage H., Tran Q., Seaman B. (2019). Neural Information Processing: 26th International Conference, ICONIP 2019.

[36] Ji S., Wang X., Zhao W., Guo D. (2019).

[37] Wang S., Yang Y. (2021). M-GAN-XGBOOST model for sales prediction and precision marketing strategy making of each product in online stores. Data Technologies and Applications.

[38] Monett D., Lemke C., Anandarajah L., Brandherm T. (2022, November). ECIAIR 2022 4th European Conference on the Impact of Artificial Intelligence and Robotics.

[39] Dannecker L. (2015).

[40] Soni Vishal Dineshkumar (2020). Emerging roles of artificial intelligence in Ecommerce (July 11, 2020). International Journal of Trend in Scientific Re- search and Development.

[41] Li L., Zhang J., Wang Y., Ran B. (2018). Missing value imputation for traffic-related time series data based on a multi-view learning method. IEEE Trans. Intell. Transport. Syst..

[42] Shahbazi H., Karimi S., Hosseini V., Yazgi D., Torbatian S. (2018). A novel regression imputation framework for Tehran air pollution monitoring network using outputs from WRF and CAMx models. Atmospheric Environ- ment.

[43] Ioffe S., Szegedy C. (2015, June). International Conference on Machine Learning.

[44] Gárate-Escamila A.K., El Hassani A.H., Andrès E. (2020). Classification models for heart disease prediction using feature selection and PCA. Informatics in Medicine Unlocked.

[45] Bakir H., Chniti G., Zaher H. (2018). E-Commerce price forecasting using LSTM neural networks. International Journal of Machine Learning and Computing.

[46] Elena P. (2021). Predicting the Movement Direction of OMXS30 Stock In- dex Using XGBoost and Sentiment Analysis.

